# Localisation of renin-angiotensin system (RAS) components in breast

**DOI:** 10.1038/sj.bjc.6603213

**Published:** 2006-06-06

**Authors:** M Tahmasebi, S Barker, J R Puddefoot, G P Vinson

**Affiliations:** 1 School of Biological Sciences, Queen Mary, University of London, Mile End Road, London E1 4NS, UK

**Keywords:** breast cancer, (pro)renin, angiotensin converting enzyme (ACE), AT1, AT2 receptors, angiotensinogen

## Abstract

Angiotensin II has mitogenic and angiogenic effects and its receptors are widespread, particularly in epithelial tissue. Tissue renin angiotensin systems (tRASs) may be a local source of angiotensin II that has specific paracrine functions. To investigate the presence of a tRAS in normal human breast and tumours. Immunocytochemistry, and quantitative RT–PCR was used to establish: (i) the presence and localisation of RAS components, (ii) the possibility of their involvement in cancer. (1) mRNA coding for angiotensinogen, prorenin, angiotensin converting enzyme (ACE), and both AT1 and AT2 receptors was demonstrated in normal and diseased breast tissues. (2) (pro)renin was identified in epithelial cells in both normal and diseased tissue, but in invasive carcinoma, its distribution was mostly confined to fibroblasts or could not be detected at all. (3) Angiotensin converting enzyme was shown in epithelial cells in both normal and malignant tissue. The results are consistent with the hypothesis that a tRAS is present in the breast, and is disrupted in invasive cancer.

The renin angiotensin system (RAS) has received most attention in relation to its functions in the circulation, in which the generation of the active hormone, angiotensin II (Ang II), is associated with the regulation of aldosterone secretion, salt and water metabolism and blood pressure (Mulrow, 1999; Kaschina and Unger, 2003). In recent years, attention has also focused on the evidence for widespread local tRASs (Mulrow and Franco-Saenz, 1996; Vinson *et al*, 1997; Tahmasebi *et al*, 1999; Nielsen *et al*, 2000; Li *et al*, 2004).

Angiotensin types 1 and 2 (AT1 and AT2) receptors are present in many different tissue types, and in particular, the AT1 receptor is abundant in secretory epithelial tissue (Vinson *et al*, 1995, 1997; Marsigliante *et al*, 1996). As Ang II and tRASs may have an important role in tissue growth and modelling these observations lead to the possibility that tRASs are involved in cancer.

While Ang II has been used in cancer to enable better accessibility of chemotherapeutic drugs to the tumour (Noguchi *et al*, 1988), Ang II blockers and ACE inhibitors have nevertheless been shown to reduce tumour size, angiogenesis and metastasis (Fujita *et al*, 2002; Uemura *et al*, 2005), although the suggestion that long-term use of angiotensin converting enzyme (ACE) inhibitors in patients may limit the development of cancer (Lever *et al*, 1998) has been questioned (Li *et al*, 2003; Ronquist *et al*, 2004).

Tahmasebi *et al* (1998) showed the transcription of (pro)renin in fibroblasts and myoepithelial cells in normal and abnormal breast tissue, suggesting the existence of a tRAS within the breast. The generation of Ang II within the breast would support the hypothesis that Ang II could directly or indirectly contribute to breast cancer progression (Inwang *et al*, 1997; Tahmasebi *et al*, 1998; De Paepe *et al*, 2001).

Using immunocytochemistry, and real time RT–PCR, the present study addresses the possibility that a tRAS exists in normal and cancerous breast tissue,

## MATERIALS AND METHODS

### Quantitative RT–PCR

Quantitative RT–PCR (QRT–PCR) was carried out using Taqman fluorogenic probe and primers specific for angiotensinogen, prorenin, ACE and the angiotensin receptors (AT1 and AT2) (Applied Biosystems, Warrington, UK). Total RNA from normal human liver, kidney and lung (BD Biosciences, Clontech, Oxford, UK) were used to set up standard curves for each gene as appropriate (five-fold serial dilutions ranging from 0.4 to 250 ng of total RNA per reaction, in triplicate). Values for normal breast and breast tumour (infiltrating ductal carcinoma) (BD Biosciences, Clontech) were obtained as anoograns RNA equivalents of the reference gene in each case derived from the corresponding standard curve. Reverse transcription and PCR amplification were performed in an MX3000P real time PCR system (Stratagene) using SuperScript ™ III one-step qRT–PCR reagent (Invitrogen, Paisley, UK) under the reaction conditions described in Table 1. Nuclease-free water was used in place of RNA template as a nontemplate control (NTC).

### Immunocytochemistry

#### Tissue collection

Formalin-fixed, paraffin wax-embedded and frozen human tissue sections

Human breast tissue samples were provided with appropriate informed consent from patients undergoing surgery. Ethical approval was granted from the Local Ethical Committee. Patients undergoing breast surgery (mean age 58 years), included normal breast tissue and benign (23 patients), premalignant and malignant breast lesions (77 patients). Paraffin wax-embedded and frozen tissues were generously provided by Dr C Brown (Department of Morbid Anatomy, Royal Hospitals Trust, London) and Ms S Jones (Department of pathology, St Bartholomew's Hospital, London) respectively.

Antibodies: The monoclonal anti-renin mouse IgG antibody 2D12 was a gift from Professor Pierre Corvol (Collège de France, Paris) and the polyclonal anti-ACE mouse IgG, from Chemicon International Inc (Hampshire,UK) Anti-vimentin (mouse IgM), anti-cytokeratin (mouse IgG) and anti-actin mouse (IgG) antibodies (Sigma, Poole, Dorset, UK) were used to discriminate between fibroblasts, epithelial cells and myoepithelium, respectively. Other reagents were from *Sigma* unless stated otherwise.

Avidin-biotin immunodetection-frozen tissue sections: Frozen tissue sections (4–8 *μ*m) were fixed with acetone on poly-L-lysine-coated slides and fixed in cold acetone, washed in three changes of Tris-buffered saline (TBS, pH 7.6, 50 mmol Tris/l, 150 mmol NaCl/l, 2 mmol MgCl_2_/l pH 7.4) and incubated for 5–10 min in hydrogen peroxide (0.1–1%) in TBS. Finally, sections were washed in TBS twice for 5 min at room temperature. Nonspecific antibody binding was blocked with 1% (w/v) normal rabbit blocking reagents (Boehringer Mannheim, Germany) and the sections were incubated with their respective primary antibodies for either 90 min at room temperature or overnight at 4°C in a humidification chamber. Negative control samples were incubated with TBS buffer alone. Unbound antibody was removed by washing 3 × for 3 min in TBS. Specific binding was detected by incubating the sections with secondary biotin-conjugated antibody, rabbit anti-mouse IgG, (1 : 300–1 : 400; DAKO, Cambridgeshire, UK) for 30 min and amplified using an avidin/streptavidin, biotinylated enzyme complex (ABC) system (DAKO) according to the manufacturers instructions followed by visualisation using diaminobenzidine hydrochloride (DAB, 3,3′-diaminobenzidine). Sections were counter-stained using Harris haematoxylin (VWR, Poole, UK) destained in acid alcohol (10 ml 1% HCl in 990 ml of 70% industrial methylated spirit (IMS; VWR). Sections were dehydrated by washing in 95% alcohol, 100% alcohol (2 × 1 min), and finally xylene, (2 × 3 min) prior to mounting in Depax mounting medium (VWR).

Avidin–biotin immunodetection-paraffin wax embedded sections: Waxed embedded tissue sections were washed in xylene, 3 × 5 min and rinsed in 100, 90 and 80% alcohol (2 × 2 min washes each). To block endogenous peroxidase activity, sections were incubated in 3% hydrogen peroxide (v/v), diluted in methanol for 15 min at room temparature. Sections were washed in distilled water for 10 min and microwaved (700 W) for 16 min in 10 mmol l^−1^ citrate buffer. Immunostaining was carried out at as previously described.

Breast cancer cell lines fixed on to the poly-L-lysine slides: MCF-7 and T47-D breast cancer cell lines were purchased from European Collection of Animal Cell Cultures (ECACC; Salisbury, UK) and routinely cultured (Puddefoot *et al*, 2002). Cells were harvested and seeded on to poly-L-lysine coated slides, then incubated at 37° under 5% CO_2_ for 24 h in a serum-free medium.

Slides were washed briefly with TBS (pH 7.6) (2 × 1 min), then fixed in cold acetone for 5 min and airdried prior to immunocytochemistry as above.

## RESULTS

### QRT–PCR

#### AT1 and AT2 receptors

**Figure 1:**
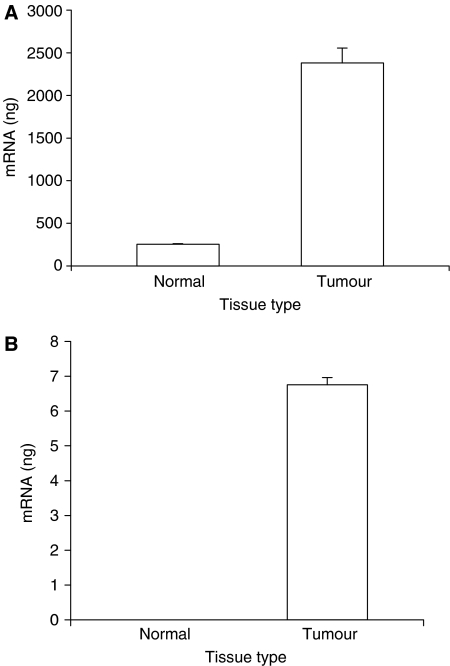
Quantitative result for AT1 (**A**) and AT2 (**B**) receptor mRNA transcription using real time reverse transcriptase–polymerase chain reaction (qRT–PCR) in normal breast tissue and infiltrating ductal carcinoma. Samples used contained 75 ng total RNA from normal breast and tumour tissue.

Values for AT1 and AT2 receptor mRNA expression were obtained by reference to a standard curve generated using normal human kidney total RNA. Normal breast tissue gave a value for AT1 receptor mRNA comparable to that found in human kidney or lung, however, the breast tumour sample showed a much higher abundance of AT1 receptor mRNA, 10-fold higher than found in normal breast tissue (Figure 1A).

AT2 mRNA was barely detectable in normal breast tissue, but it was clearly present in breast tumour, *albeit* at lower levels than in either kidney or lung (Figure 1B). Nontemplate control tubes gave no detectable amplification.

#### RAS components

**Figure 2:**
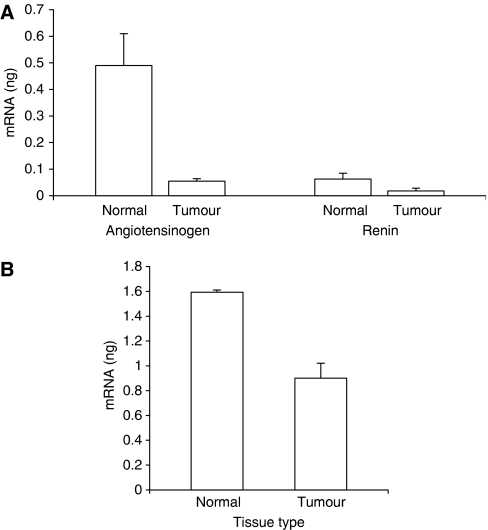
Quantitative result for angiotensinogen, prorenin and ACE mRNA transcription using real time reverse transcriptase–polymerase chain reaction (qRT–PCR) in normal breast tissue and infiltrating ductal carcinoma. Samples used contained 250 ng total RNA from normal breast and tumour tissue for angiotensinogen and prorenin (**A**), and 75 ng total RNA from normal breast and tumour tissue for ACE (**B**).

Values for angiotensinogen (AGT), prorenin and ACE mRNA expression were obtained by reference to standards curves generated using normal human liver, kidney and lung total RNA, respectively. AGT mRNA was detected at relatively low, although significant levels in both normal breast and in breast tumour samples. Nontemplate control tubes gave no detectable amplification. There was no significant difference between the levels of AGT mRNA found in normal and breast tumour tissue (Figure 2A).

Prorenin mRNA was also present in both normal breast and breast tumour samples and although values were relatively low, significantly higher levels were detected in normal compared to breast tumour tissue (Figure 2A).

ACE mRNA was also lower in tumour tissue compared with normal tissue (Figure 2B). In both cases, nontemplate control tubes gave no detectable amplification.

#### Immunocytochemistry

Cellular identification was confirmed by reference to slides immunostained for cytokeratin (epithelial cells), actin (myoepithelial cells), and vimentin (fibroblasts) (results not shown).

#### (pro)renin

**Figure 3:**
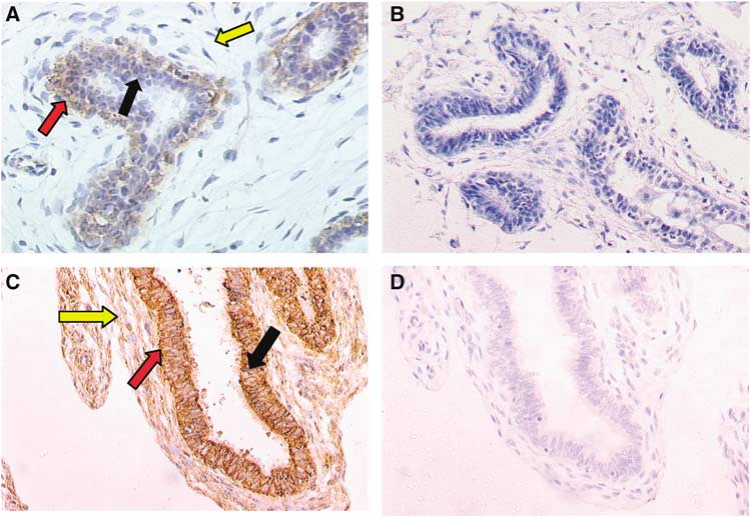
(Pro)renin was localised in sections of formalin-fixed, paraffin-embedded normal breast tissue. (**A**) Immunoperoxidase staining shows the antigen (pro)renin was present in myoepithelial cells (red arrow). Staining was absent from epithelial cells (black arrow) and connective tissues (yellow arrow). In fibroadenoma (**C**), immunoperoxidase staining shows the (pro)renin was present in epithelial cells, myoepithelial cells, and connective tissues. Control sections, omitting primary antibody, showed no immunostaining (**B** & **D**). Magnification × 200.

In normal tissue sections, immunoreactive (pro)renin was shown to be distributed almost exclusively in myoepithelial cells (Figure 3A), and connective tissue while epithelial cells showed virtually no staining. In contrast, in fibroadenoma tissue, (pro)renin was now found to be localised in epithelial cells (Figure 3C), as well as in myoepithelial cells, and stromal fibroblasts and connective tissue. Control sections in the absence of primary antibody (Figure 3B and D) showed no immunostaining.

In malignant tumours, most of the (pro)renin staining was seen in myoepithelial cells with, some patchy staining in epithelial cells, and also in fibroblasts.

**Figure 4:**
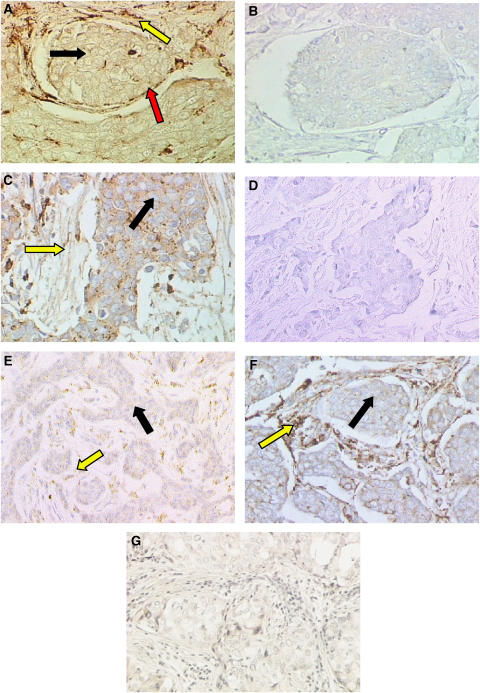
(Pro)renin was localised in sections of formalin-fixed, paraffin-embedded ductal carcinoma *in situ*. (**A**) Immunoperoxidase staining shows the antigen was present in myoepithelial cells (red arrow) and fibroblasts (yellow arrow). Staining was absent from epithelial (cancer) cells (black arrow). In infiltrating ductal carcinoma grade II (**C**) (pro)renin was present in epithelial cells (black arrow). Staining was absent from majority of the fibroblasts (yellow arrow). In infiltrating ductal carcinoma grade III (**E**), the antigen was present in fibroblasts (yellow arrow). Staining was absent from epithelial cells (black arrow). In lobular carcinoma *in situ* (**F**) the antigen was present in fibroblasts (yellow arrow) with a very weak staining in epithelial cells (black arrow). Control sections (**B**), (**D**), and (**G**), omitting primary antibody, showed no staining. Magnification × 200.

In ductal carcinoma *in situ*, positive (pro)renin staining was observed in the myoepithelial cells and fibroblasts as a disrupted band surrounding the ducts. The staining varied from a uniform and usually strong staining of most cells at intensity equivalent to that seen in normal tissues to rather patchy, less positive staining (Figure 4A). Nevertheless, (pro)renin expression was present in all cases. Epithelial cells showed no immunoreactivity. The staining reaction was abolished in the absence of primary antibody (Figure 4B). In infiltrating ductal carcinoma grade II, (pro)renin staining was absent from the majority of fibroblasts whereas some epithelial cells showed positive staining (Figure 4C). When present, myoepithelial cells showed positive staining. Control sections, omitting primary antibody showed no immunostaining (Figure 4D).

In grade III intraductal carcinoma, the cancer cells show little (pro)renin staining, and instead, the fibroblasts frequently show the greatest abundance of the antigen. The pattern of staining was variable, and ranged within individual sections from uniform and strong, to a more patchy distribution with weak or moderate staining which did not reach the intensity seen in normal tissues. However, in some cases of poorly differentiated invasive carcinoma, epithelial cells showed some immunoreactivity with only a weak, patchy staining in fibroblasts (Figure 4E).

In lobular carcinoma *in situ*, positive staining for (pro)renin was observed in myoepithelial cells and connective tissues surrounding the lobules (Figure 4F). A very weak stain was shown in the cytoplasm of the epithelial cells. The negative controls in the absence of primary antibody showed no staining (Figure 4G).

**Figure 5:**
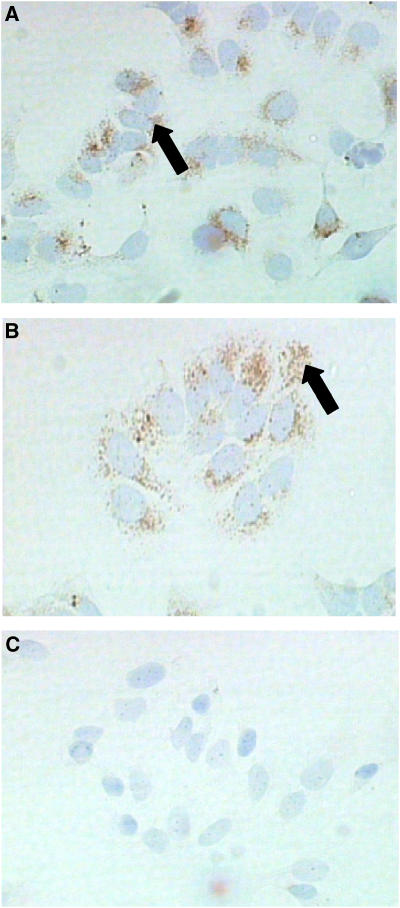
Immunoperoxidase staining of methanol-fixed MCF-7 (**A**) and T47-D (**B**) breast cancer cells. (Pro)renin was present in both (black arrow). There was no staining in the absence of primary antibody, shown here for MCF-7 cells (**C**). Magnification × 200.

Both MCF-7 (Figure 5A) and T47-D (Figure 5B) cell lines showed strong prorenin expression. Control sections for MCF-7 cell line, gave no staining in the absence of primary antibody (Figure 5C).

#### ACE

These studies were carried out on frozen sections because the primary antibody used was suitable for frozen sections, but not for paraffin-embedded sections.

**Figure 6:**
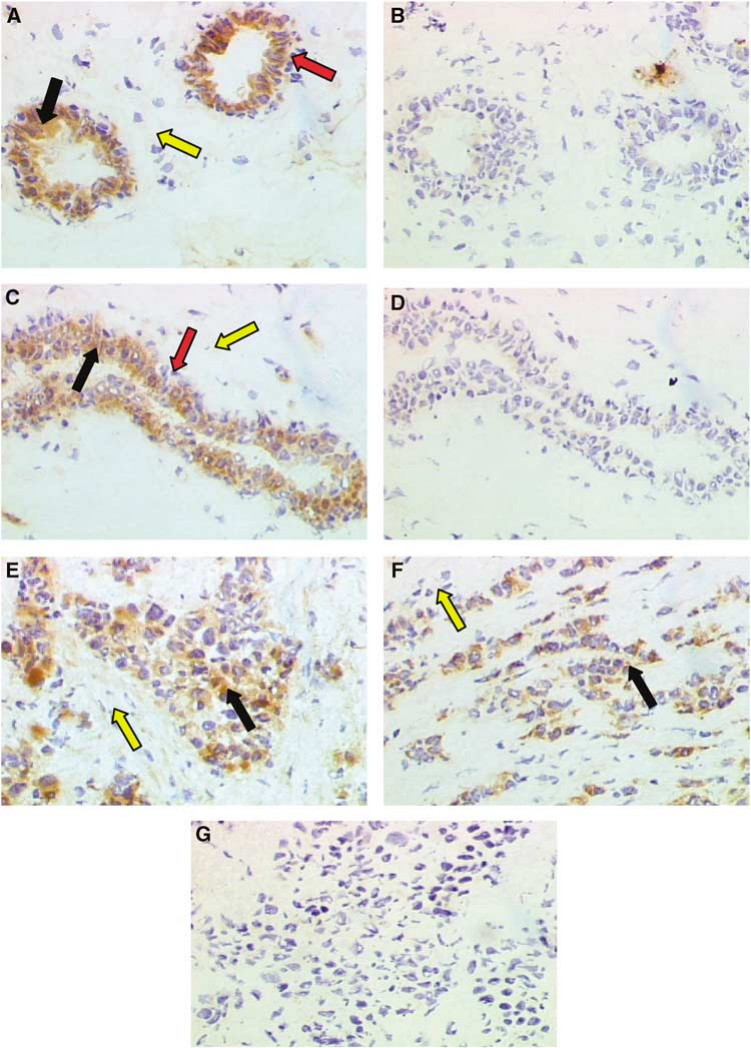
ACE was invariably localised in epithelial cells in frozen sections of all tissue examined, illustrated here for normal breast tissue (**A**) fibroadenoma (**C**) infiltrating ductal carcinoma grade III (**E**, **F**). Staining was absent from myoepithelial cells (red arrows) and connective tissues (yellow arrows). Control sections, omitting primary antibody, showed no immunostaining (**B**, **D**, **G**). Magnification × 200.

The results show that ACE was present in epithelial cells of normal breast and virtually all the epithelial cells were positively stained (Figure 6A). In all cases fibroblasts were negative. The negative control shows no staining in the absence of the primary antibody (Figure 6B).

In fibroadenoma too, epithelial cells, stained positively for ACE (Figure 6C).

The connective tissues surrounding the ducts were completely negative. The negative control shows no staining in the absence of the primary antibody (Figure 6D).

In invasive ductal carcinoma grade III, ACE was expressed in the epithelial cells but apparently less uniformly strong than in normal tissue (Figure 6E and F). The staining intensity ranged from strong in benign cells to moderate in poorly differentiated cancer cells. The negative control shows no staining in the absence of the primary antibody (Figure 6G). In all cases the connective tissue showed no immunostaining.

## DISCUSSION

It is increasingly clear that the the functions of the RAS extend beyond its roles in sodium and potassium homeostasis and the regulation of blood pressure and many studies have shown that it has a wider significance. For example, Ang II also has trophic and apoptotic activities in various cell types (Bedecs *et al*, 1997; Dinh *et al*, 2001; Kaschina and Unger, 2003; Li *et al*, 2005). Furthermore, immunolocalisation of the AT1 receptor also strongly suggests that Ang II has a widespread role in maintenance of epithelial structure and function (Vinson *et al*, 1995, 1997). Such functions may include the regulation of water and electrolyte transport (Vinson *et al*, 1995; Quan and Baum, 1996) as well as mitosis and tissue differentiation (Bedecs *et al*, 1997; Li *et al*, 2005).

As Ang II and tRASs may have an important role in epithelial tissue growth and modeling, it is possible that they may be involved in cancer.

There is clear evidence for the presence of Ang II receptors in different types of cancer. (Marsigliante *et al*, 1996; De Paepe *et al*, 2001; Deshayes and Nahmias, 2005; Suganuma *et al*, 2005). In breast tissue, both AT1 and AT2 receptors are present, and the AT1 is located in both lobular and ductal epithelial cells in normal and benign breast tissues (Inwang *et al*, 1997), and increased AT1 receptor mRNA transcription has been shown in cancer as compared with normal cells (Greco *et al*, 2002a), although receptor expression may decrease in invasive carcinoma, compared with hyperplasia or ductal carcinoma *in situ* (De Paepe *et al*, 2001). In addition, AII has been shown to have an influence on breast cancer cell metastasis throught its control of integrin expression (Puddefoot *et al*, 2006). From this evidence, it is a clear possibility that Ang II may be involved in the maintenance of normal breast and epithelial tissue structure, and *in vitro* studies have shown stimulation of proliferation in primary breast tissue cultures via the AT1 receptor, together with the importance of the PKC pathway involved (Greco *et al*, 2002b; Muscella *et al*, 2002).

There is evidence too for the existence of tRASs in cancer. The expression of angiotensinogen, (pro)renin, ACE, and AT1 and AT2 receptors has been demonstrated in glioblastoma tumours and glioblastoma cells in culture, in which renin has a direct role in proliferation and/or survival (Juillerat-Jeanneret *et al*, 2004). In an earlier study from this laboratory, (pro)renin gene transcription was demonstrated in normal and abnormal breast tissue using *in situ* hybridisation (Tahmasebi *et al*, 1998). (Pro)renin transcription was found in nearly all samples, but there were differences between normal and abnormal tissue. In normal breast ducts, transcription was seen in myoepithelial cells and in fibroblasts, but none was found in the secretory epithelium. In cancer, overall renin transcription was seemingly reduced with the loss of myoepithelial cells, and it also became more sporadic in fibroblasts.

To extend these studies, the present work was designed first to confirm the presence of AT1 and AT2 receptor mRNA, together with mRNA coding for components of the RAS. Quantitative RT–PCR analysis revealed the transcription of AT1 and AT2 receptor mRNA in both normal and diseased human breast tissues though AT1 mRNA was much more abundant in carcinoma than in normal tissue (Figure 1A and B), thus supporting the growing body of evidence discussed previously that increased AT1 receptor expression may contribute to mammary carcinoma (Greco *et al*, 2002a). Angiotensinogen mRNA was present in very low amounts compared with the liver and prorenin mRNA was present although lower than in the kidney (Figure 2A), unsurprisingly, since liver and kidney are usually considered to be the major sources of these components (Mulrow, 1999; Kaschina and Unger, 2003). However, confirming the previously reported *in situ* hybridisation data (Tahmasebi *et al*, 1998), there was significantly less (pro)renin mRNA in carcinoma than in normal tissue. Finally, the quantification of ACE mRNA showed that expression was present in carcinoma, although again in lower amounts than in normal tissue (Figure 2B).

This demonstrates that RAS components may be produced in the breast, and leads to the question of their localisation.

In the present study, immunocytochemistry revealed that, in most samples of normal breast tissue, (pro)renin was in present in abundance in myoepithelial cells but, unlike its mRNA, it was absent from connective tissues surrounding the ducts (Figure 3A). This was also true in ductal and in lobular carcinoma *in situ* (Figure 4A and F). Different staining patterns were seen in fibroadenoma (Figure 3C), and in infiltrating ductal carcinoma (Figure 4C) in which the antigen was weakly and sporadically present in epithelial cells, but also more strikingly in fibroblasts as well. Abundance of (pro)renin also varied according to the stage of malignancy, suggesting that its expression varied inversely with tumour grading. Thus, (pro)renin staining was still present in fibroblasts, although at relatively low intensity (Figure 4E).

Together with previous data on the distribution of mRNA, which in normal tissue was detected mostly in fibroblasts and myoepithelium, the data suggest that (pro)renin, is formed in the fibroblasts (and myoepithelium) and is transported from the connective tissue site of synthesis, perhaps to myoepithelium (although the myoepithelium is also a source), and possibly even to the epithelium, though this is has only been faintly visible in some of the abnormal samples (Figure 4C). In cancer, although, (pro)renin mRNA and protein are both found in fibroblasts. This suggests that the intimate association between fibroblasts, myoepithelium and epithelium are essential for any such transfer to occur. Together with the progressive loss overall of both (pro)renin mRNA and protein, such physical disruption suggests that the system for local Ang II generation is greatly impaired in cancer.

The localisation of ACE is especially important to the concept of a local RAS supplying purely local requirements for Ang II. The immunohistochemical results presented here shows that ACE is largely distributed in the epithelial cells in human benign and malignant breast tissues (Figure 6A, C, E and F). This distribution matches that of the AT1 receptor, but is different from that of (pro)renin. It suggests that Ang II in breast tissue is not necessarily derived from the circulation and may originate from a local source. Most importantly, it suggests that Ang II is produced directly in the epithelium, the site at which it acts. Clearly it may be formed from Ang I provided by renin activity in the myoepithelium. This reveals a tightly organised RAS that is geared to the production of hormone in the breast duct epithelium alone.

It is the apparently close coupling of (pro)renin and ACE expression with the epithelial site of Ang II action that is so compelling about the data presented here. As Ang II has numerous functions in epithelial and other tissues, including the regulation of mitosis and tissue differentiation, the observation that (pro)renin transcription apparently fails in invasive carcinoma, crucially suggests that here Ang II is not available to maintain these functions. This has profound implications for our understanding of cancer. We conclude that the local tRAS may be important for the regulation of epithelial function in the breast. Such regulation is lost as the renin producing fibroblasts and myoepithelium are physically separated from the bulk of the proliferating cells. The characteristic invasiveness of malignant cells may reflect this loss of RAS control.

## CONCLUSION

It is known that Ang II has a role in tissue structure and cell proliferation, and its proliferative actions have been shown in breast cancer cells. It is also known that the ductal epithelium is abundantly supplied with AT1 and receptor, and, from the present work, also with ACE. From previous and from the present work, it is also now known that the epithelium is surrounded by fibroblasts and myoepithelial tissue that transcribe and express (pro)renin, and angiotensinogen mRNA is also present. These findings show the possibility of a tRAS in the breast that is tightly coupled to the site of action of the active hormone, Ang II. This system is disrupted in cancer, leading to the possibility that failure of the RAS, and Ang II production may contribute to the functional phenotype of cancer cells.

A better understanding of the progression-specific alterations in tRAS components in breast cancer could therefore be helpful in the development of targeted therapies.

## Figures and Tables

**Table 1:** Thermal cycling parameters for qRT–PCR

**QRTPCR parameters**			
		**PCR amplification cycles**	
**Initial steps**		**Melt**	**Anneal/extend**
30 min	10 min	15 s	30 s
50°C	95°C	95°C	60°C
